# The ability of pentose pathways to form all essential metabolites provides clues to the origins of metabolism

**DOI:** 10.1371/journal.pbio.3002996

**Published:** 2025-01-10

**Authors:** Steffen N. Lindner, Markus Ralser

**Affiliations:** 1 Department of Biochemistry, Charité Universitätsmedizin Berlin, corporate member of Freie Universität Berlin and Humboldt-Universität, Berlin, Germany; 2 Max Planck Institute for Molecular Plant Physiology, Potsdam-Golm, Germany; 3 Centre For Human Genetics, University of Oxford, United Kingdom; 4 Max Planck Institute for Molecular Genetics, Berlin, Germany

## Abstract

The structure of the early metabolic network is unknown. Here, we report that when considered together, pentose utilization pathways form all life-essential precursors. We speculate that the chemistry preserved in pentose metabolism could therefore have been a central structural element in early metabolism.

The remarkable conservation of metabolic networks across all living systems suggests that the fundamental structure of metabolism was established during the earliest stages of life on Earth, which emerged at least 4 billion years ago. In modern organisms, the metabolic network spans over hundreds of enzymes turning over thousands of different metabolites [1]. In this network, “catabolic” and “anabolic” reactions, i.e., reactions which build up or break down metabolites, are intertwined, which implies that early metabolism contained features of both heterotrophy (i.e., the consumption of extracellular metabolites) and autotrophy (i.e., the self-synthesis of these metabolites). Among competing hypotheses about the origin of this conserved, basic structure, there is a growing view that this network emerged from non-enzymatic reaction sequences, at least in part driven by iron (Fe(II)) and other metal ion catalysts [2–4]. In this model, early enzymes were already amino acid-based and were selected by accelerating and increasing the specificity of the non-enzymatic reactions [5–8]. A model that places non-enzymatic reaction sequences at the root of the metabolic network also overcomes the “end product problem,” which describes that multistep, enzymatic reaction sequences need a precursor to evolve: enzymes that form intermediates cannot be selected by Darwinian evolution directly, as they provide no advantage without the downstream steps that form the functional, and therefore selectable, “end”-product [9].

Indeed, evidence about the role of metal ions in metabolic evolution is growing. Even in the absence of enzymes, metal ions drive metabolism through interconversion reactions such as those participating in glycolysis, the pentose phosphate pathway (PPP), and the oxidative and reductive Krebs cycles [2,3]. Recent studies have shown that approximately 90% of metabolic pathways depend on metal ions in modern organisms [10], that iron is recovered as the most abundant transition metal in Archean sediment [11], and that amino acids such as cysteine can catalyze central metabolic reactions like the formation of ribose 5-phosphate [6,8].

Examining the structure of this extant metabolic network closely, one notices that the vast majority of essential biosynthetic pathways branch from a limited number of precursors such as glucose-6-phosphate, pyruvate, and succinyl-CoA, among others. The conservation of the metabolic network suggests an early metabolic structure that provided these intermediates. In many modern organisms, these metabolites are interconverted by a combination of gluconeogenesis and/or glycolysis (and their variants), the PPP and the Krebs cycle. Certainly, their favorable properties such as rate, efficiency, and little carbon loss, contributed to the success of these pathways in evolution. However, the centrality of hexose interconversions for early metabolism remains unclear. For instance, the Embden–Meyerhof–Parnas (EMP) and the Entner–Doudoroff (ED) (glycolytic) pathways, as well as their alternatives, convert C_6_ sugars into C_3_ metabolites, ultimately yielding pyruvate. However, these pathways require multiple reactions (e.g., 10 for EMP and ED) and lack direct connections to the oxidative Krebs cycle.

Pentoses are equally abundant in nature. While glucose is a major storage and structural component of photosynthetic organisms, as components of sugar polymers like cellulose, amylose, and glycogen, pentoses such as xylose and arabinose are major hemicellulose components. Moreover, energy stored in glucose and pentoses can be used to generate ATP via fermentation in substrate-level phosphorylation under anaerobic conditions. However, while hexoses themselves are mostly important transport and storage forms for carbohydrates, pentoses also play a direct role in metabolism; they form the sugar-phosphate backbone of RNA and DNA, and are structural components of nucleotides, such as ATP, NAD(P), and FAD.

Our interest in pentose metabolism was further sparked by the huge variety of pentose utilization pathways, contrasting with the uniform structure of hexose and citrate pathways. To delve deeper into the complexity of pentose utilization, we overlaid the pathway maps with the widely used essential biomass precursor molecules (Fig 1). The pathways of pentose utilization can be categorized into 3 groups: those utilizing xylose directly, those starting from a phosphorylated pentose derivative, and those utilizing pentose-phosphates with a simple C_1_ co-substrate. Following these categories, we examined these pathways in greater detail, emphasizing their metabolic products in the context of generating essential biomass precursor molecules.

**Fig 1:**
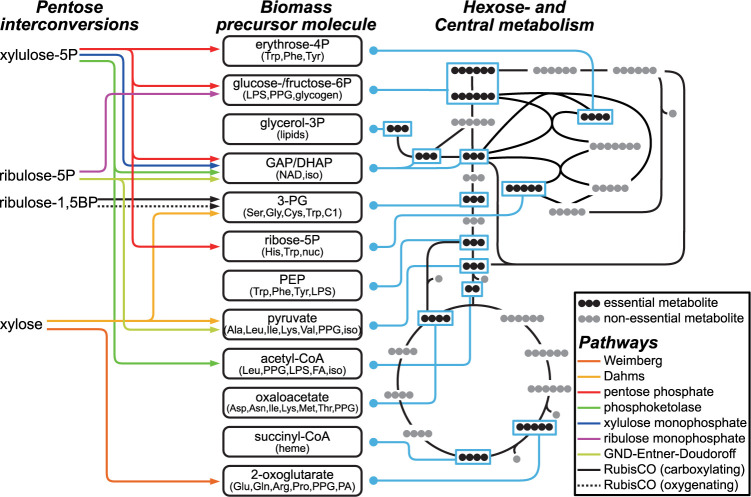
Pentose utilization pathways include all life-essential metabolic precursors as intermediates. Essential biomass precursors as intermediates of canonical central metabolism routes (right) are displayed in black dots (corresponding to the number of carbon atoms) and highlighted by blue frames. The Dahms pathway generates GA (feeding via the amination of glyoxylate or the glycerate pathway into Ser and Gly biosynthesis, and via glyoxylate shunt into Asp family amino acid biosynthesis) and pyruvate (a precursor for Ala, Ile, Leu, Val, Lys, and PPG synthesis). The Weimberg pathway generates 2-oxoglutarate (a precursor of Glu family amino acids). The non-oxidative PPP generates fructose 6-P (with glucose 6-P, a precursor of PPG, LPS, and glycogen) and GAP (a precursor of iso, GAP can be converted to DHAP: heme, NAD, and lipids). PKT generates GAP and acetyl-phosphate which can be converted to acetyl-CoA: leu, FA, iso. With formaldehyde, RuMP generates fructose 6-phosphate and XuMP generates GAP and DHAP. With CO_2_ RubisCO generates 3-phosphoglycerate (Ser and derived compounds). With O_2_ RubisCO generates 3-phosphoglycerate and 2-PG, a precursor of glyoxylate. With CO_2_ the GED pathway generates GAP and pyruvate. Ala, alanine; Arg, arginine; Asn, asparagine; Asp, aspartate; C1, one carbon containing compounds; Cys, cysteine; DHAP, dihydroxyacetone-phosphate; FA, fatty acids; GA, glycolaldehyde; Gln, glutamine; Glu, glutamate; Gly, glycine; His, histidine; Ile, isoleucine; iso, isoprene; Leu, leucine; LPS, lipopolysaccharides; Lys, lysine; Met, methionine; NAD, nicotine adenine dinucleotide; nuc, nucleotides; 2-PG, 2-phosphoglycolate; PA, polyamines; PEP, phosphoenol-pyruvate; Phe, phenylalanine; PPG, peptidoglycan; Pro, proline; Ser, serine; Trp, tryptophan; Tyr, tyrosine; Val, valine.

The Dahms and Weimberg pathways utilize xylose directly without prior phosphorylation of the sugar. The Dahms pathway [12] generates glycolaldehyde and pyruvate as its final products. Pyruvate is the product of glycolysis and stands at the interception between glycolysis and the Krebs cycle, essential for the biosynthesis of several amino acids. Meanwhile, glycolaldehyde can generate several intermediates of metabolism, including C_4_ intermediates of the Krebs cycle (via glyoxylate), phosphoglycerate (which enters glycolysis), and serine family amino acids (via amination). The Weimberg pathway shares some activities with the Dahms pathway but converts xylose into 2-oxoglutarate, an intermediate of the Krebs cycle [13]. Other pentose utilization routes use phosphorylated pentoses. Canonically, pentose-phosphates are utilized via the non-oxidative PPP [14], generating glycolysis intermediates fructose 6-phosphate and glyceraldehyde 3-phosphate (GAP) as its final products. Phosphoketolase (PKT) catalyzes xylulose 5-phosphate cleavage into GAP and acetyl-phosphate (acetyl-P), providing carbon for glycolysis and the Krebs cycle.

Moreover, when considering pathways capable of pentose co-utilization with CO_2_ or the simple C_1_ compound formaldehyde, both abundantly present in early Earth’s atmosphere, several additional routes add to the list of optional pentose utilization pathways. Two pathways use a pentose-phosphate as acceptor for formaldehyde assimilation: the ribulose monophosphate (RuMP) pathway, which uses ribulose 5-phosphate and formaldehyde to generate the glycolysis intermediate fructose 6-phosphate, and the xylulose monophosphate (XuMP) pathway, which generates the glycolytic C_3_ compounds GAP and DHAP from xylulose 5-phosphate and formaldehyde.

The CO_2_ assimilation reaction of ribulose bisphosphate carboxylase/oxygenase (RubisCO) uses ribulose 1,5-bisphosphate and CO_2_ to generate 2 molecules of glycolytic intermediate 3-phosphoglycerate. Additionally, RubisCO possesses an oxygenation activity, converting ribulose 1,5-bisphosphate to 3-phosphoglycerate and 2-phosphoglycolate, which can be converted to glyoxylate. Finally, at elevated CO_2_ concentrations, a prerequisite present in early Earth’s atmosphere, 6-phosphogluconate dehydrogenase (Gnd), catalyzes the reductive carboxylation of ribulose 5-phosphate to 6-phosphogluconate, which generates GAP and pyruvate via the reactions of the ED pathway.

Overlaying the pentose utilization pathways (Fig 1), one obtains a chemical network that contains the compendium of essential metabolic precursors required for amino acid, nucleotide, and fatty acid biosynthesis, as integral intermediates. This network misses only a few essential metabolites, which can be derived from network intermediates using very few reactions, like succinyl-CoA from 2-oxoglutarate, and PEP and oxaloacetate from pyruvate.

Thus, the life-essential precursors are intrinsic to the pentose interconversion pathways. It follows that the chemistry which is preserved in the compendium of pentose utilization pathways seems to have been intrinsically capable of forming the life essential precursors as required in an early metabolism. It is thus worth speculating that, despite being equally ancient, the pentose rather than the much more complex hexose interconversion pathways, could have been the central structural element shaping early metabolism, and that the favorable chemical properties of glucose and hexose interconversions are what led them to become the central metabolic pathways due to Darwinian evolution. Our observation should not be misinterpreted as a claim that the metabolic network must have started as a large pentose interconversion network, as a claim that early metabolism must have been of heterotrophic nature, or that hexose pathways would be of no importance for an early metabolism. The observation, however, generates testable hypotheses to address the origins of metabolism; for instance, that pentose interconversion sequences would need to be enabled by a non-enzymatic, metal, or amino acid promoted proto-biochemistry, which favors the selection of enzymatic catalysts.
